# Dose-Dependent Impact of Metformin on Osteoblast-Specific Biomarkers in Cultured Rat Primary Osteoblasts

**DOI:** 10.33549/physiolres.935714

**Published:** 2026-02-01

**Authors:** Monika MARTINIAKOVA, Anna SAROCKA, Vladimira MONDOCKOVA, Noemi PENZES, Veronika KOVACOVA, Roman BIRO, Radoslav OMELKA

**Affiliations:** 1 Department of Zoology and Anthropology, Faculty of Natural Sciences and Informatics, Constantine the Philosopher University in Nitra, Nitra, Slovak Republic; 2 Department of Botany and Genetics, Faculty of Natural Sciences and Informatics, Constantine the Philosopher University in Nitra, Nitra, Slovak Republic

**Keywords:** Metformin, Osteoblasts, Bone health, *In vitro*

## Abstract

The objective of this *in vitro* study was to examine the impact of metformin (MET) at different concentrations (0.1, 1, 10, 50, and 100 mM) on rat primary osteoblasts, as the results obtained so far are inconsistent. Osteoblast apoptosis, viability, alkaline phosphatase (ALPL) activity, production of osteoblast-specific biomarkers, including ALPL, osteocalcin (BGLAP), type I collagen alpha 1 (COL1A1), integrin-binding sialoprotein (IBSP), bone morphogenetic protein 2 (BMP2), runt-related transcription factor 2 (RUNX2), vascular endothelial growth factor (VEGF), tumor necrosis factor ligand superfamily member 11 (TNFSF11 or RANKL), as well as calcium/collagen deposition were assessed. Our results revealed that a dose of 100 mM was cytotoxic to osteoblasts and resulted in a complete loss of their viability. Therefore, this concentration was excluded from further analyses. In general, MET exhibited a dose-dependent impact on multiple osteoblast-specific functional biomarkers, with beneficial effects noted on ALPL activity (at 0.1 and 1 mM) as well as on the levels of ALPL (0.1 and 1 mM), BGLAP (at 0.1–50 mM), IBSP (at 0.1–50 mM), BMP2 (at 0.1, 10 and 50 mM), VEGF (at 0.1 and 1 mM), and RANKL (at 0.1 mM). Calcium/collagen deposition at concentrations of 0.1 and 1 mM reached the same level as control cells, higher doses (10 and 50 mM) dramatically reduced cell viability after 21 days and the aforementioned parameters could not be evaluated. It can be concluded that MET at concentrations up to 1 mM can promote osteoblast viability, osteogenic differentiation, angiogenic signaling, and reduce osteoclastogenesis.

Metformin (MET, 1,1-dimethylbiguanide) is the first-line oral antidiabetic medication often used to treat type 2 diabetes mellitus (T2DM) due to its ability to enhance peripheral glucose uptake, decrease hepatic glucose synthesis, and improve insulin sensitivity [1,2]. In addition to its antidiabetic benefits, MET has been shown to exhibit pleiotropic effects, such as antioxidant, anti-inflammatory, cardioprotective, anticancer, and bone protective actions [3,4]. In general, activation of AMP-activated protein kinase (AMPK), regulation of mitochondrial function, modification of reactive oxygen species (ROS) and subsequent impacts on cellular metabolism and differentiation pathways are primarily responsible for the mechanisms of its action [5,6].

Emerging evidence suggests that MET can improve bone health. Various studies performed *in vitro* show that MET inhibits osteoclastogenesis while promoting osteoblast differentiation from mesenchymal stem cells (MSCs) or embryonal cells such as MC3T3-E1 or osteoblast-like cancer cell lines (e.g. U2OS, MG63) [7–9]. However, research investigating the impact of MET on osteoblast-specific biomarkers in primary osteoblast cell cultures is rare and the results obtained are inconsistent. The majority of studies [10–12] demonstrated a protective effect of MET (at 0.1–0.8 mM) against osteoblast apoptosis and its inhibitory impact on osteoclast differentiation. The study by Patel *et al.* [13] revealed no effect of MET (at 0.1 mM) on osteoblast or osteoclast function. In contrast, Kasai *et al.* [14] pointed out its negative impact (at 0.5–5 mM) on osteoblast differentiation. The aim of this *in vitro* study was therefore to evaluate the effects of MET at different concentrations on a broader spectrum of osteoblast-specific functional biomarkers, including cell viability, apoptosis, ALPL activity, production of alkaline phosphatase (ALPL), osteocalcin (BGLAP), type I collagen alpha 1 (COL1A1), integrin-binding sialoprotein (IBSP), bone morphogenetic protein 2 (BMP2), runt-related transcription factor 2 (RUNX2), vascular endothelial growth factor (VEGF), tumor necrosis factor ligand superfamily member 11 (TNFSF11 or RANKL), as well as calcium/collagen deposition in primary rat osteoblast cell culture.

Calvarial rat primary osteoblasts (InnoProt, Rat Osteoblasts, P10931, Derio, Spain) were cultured in alpha-MEM medium (Sigma-Aldrich, St. Louis, MO, USA) supplemented with antibiotics (1 % penicillin-streptomycin solution, HyClone, Logan, UT, USA) and 10 % fetal bovine serum (FBS; Sigma-Aldrich). Cells were cultured at 37 °C in a 5 % CO_2_ atmosphere in 75 cm^2^ culture flasks until 80–90 % confluence was reached. Culture medium was changed every 2 days. Subculture was carried out according to Taylor *et al.* [15]. All experimental analyses were performed during the exponential growth phase (after the third passage). Osteoblasts were incubated without (control group) and with MET (Metformin hydrochloride, MedChemExpress, New Jersey, USA). The MET was dissolved in culture medium at final concentrations of 0.1, 1, 10, 50, and 100 mM and the cells were treated for 72 h. Osteoblast apoptosis was evaluated using the TUNEL assay and DNA fragmentation was quantified using the HT TiterTACS™ Apoptosis Detection Kit (Trevigen, Gaithersburg, MD, USA). The proportion of DNA-fragmented (apoptotic) cells was calculated as a percentage relative to the total cell number. Cell viability was assessed by the colorimetric MTS assay (CellTiter 96® AQueous One Solution Assay, Promega, Madison, WI, USA) and the results were expressed as a percentage of optical density (OD) relative to the control group. ALPL activity was detected using BCIP®/NBT SIGMAFAST™ substrate, which stains cells blue-violet in the presence of ALPL activity. Concentrations of osteoblast-specific biomarkers, including ALPL, COL1A1, IBSP, BGLAP, BMP2, RUNX2, VEGF, RANKL were determined using ELISA kits (cat. no. ER0728, ER0850, ER0777, ER1205-HS, ER0010, ER1313, ER0069, ER1604; FineTest, Wuhan, China). In a mineralization assay, treated and control cells were cultivated in osteogenic medium for 21 days. Quantification of calcium deposits was performed using the Alizarin Red S Staining Quantification Assay (Cat. no. 8678, ScienCell Research Laboratories, USA). Collagen deposition was calculated from absorbance measurements after culturing osteoblasts in alpha-MEM medium for 21 days and staining the cells with 0.1 % Sirius Red in saturated aqueous picric acid. Images of stained cells were captured using an inverted light microscope at original magnification ×100. Statistical analysis was performed using SPSS v. 26.0 software (IBM Corp., Armonk, NY, USA). The results were presented as mean ± standard deviation (SD) of experiments measured as triplicate with three to eight technical replicates. The data obtained were evaluated using one-way ANOVA with Games-Howell and/or Tukey’s *post hoc* tests. P values lower than 0.05 were considered statistically significant.

The highest concentration of MET (100 mM) was cytotoxic to osteoblasts and resulted in a complete loss of their viability. Therefore, this concentration was excluded from further analyses. A dose of 1 mM significantly enhanced cell viability, the remaining doses reached the level of control cell viability. MET at concentrations ranging from 0.1 to 10 mM did not affect osteoblast apoptosis, but a dose of 50 mM considerably reduced it. Considering ALPL activity, a biphasic MET response was observed, with lower doses (0.1 and 1 mM) increasing ALPL activity, while higher doses (10 and 50 mM) leading to its decrease. MET elevated the production of ALPL (at 0.1 and 10 mM), BMP2 (at 0.1, 10, and 50 mM), VEGF (at 0.1 and 1 mM), BGLAP (at 0.1–50 mM), and IBSP (at 0.1–50 mM). These findings demonstrate that MET can promote osteoblast differentiation and function, as well as angiogenic signaling. Conversely, MET downregulated RANKL (at 0.1 mM), indicating reduced osteoclastogenesis. Higher concentrations of MET (10 and 50 mM) also decreased COL1A1 levels, reflecting increased enzymatic degra-dation and/or decreased secretion of type I collagen. None of the MET doses used affected RUNX2 levels (Fig. 1). RUNX2 is a master regulator of bone development, which is highly expressed especially in immature osteoblasts and its amount decreases in mature osteoblasts. Furthermore, the concomitant upregulation of its downstream targets including ALPL, BGLAP, IBSP suggests that MET may enhance osteogenesis through post-translation activation of RUNX2, primarily by elevating its phosphorylation by AMPK, rather than by increasing RUNX2 levels [16]. Calcium/collagen deposition at concentrations of 0.1 and 1 mM reached the same level as control cells (Fig. 2), higher doses (10 and 50 mM) dramatically reduced cell viability after 21 days with minimal numbers of live osteoblasts. Consequently, the aforementioned parameters were not assessed at these doses.

In *in vitro* experiments, MET is often applied at concentrations ranging from μM to mM, with tens of μM typically achievable in plasma under *in vivo* conditions. However, higher doses may still provide valuable insights into underlying mechanisms or threshold effects, as demonstrated in several studies [14,17]. Our results showed that MET exhibited a dose-dependent impact on multiple osteoblast-specific biomarkers, including ALPL activity, as well as ALPL, BGLAP, IBSP, BMP2, VEGF, RANKL levels, with beneficial effects at lower concentrations (especially at 0.1 and 1 mM). In this context, Duan *et al.* [17] discovered that higher doses of MET (5 and 10 mM) impaired viability and induced apoptosis of MSCs *in vitro*. It is noteworthy that in our research, concentrations of 10 and 50 mM did not significantly reduce osteoblast viability and even the 50 mM dose decreased their apoptosis, which could support the fact that MET (50 mM) can promote cell differentiation through the regulation of autophagy and ROS [18]. In contrast to Kasai *et al.* [14], who found no differences in rat primary osteoblast viability after MET treatment at doses ranging from 0.5 to 5 mM, we observed enhanced osteoblast viability at 1 mM. Consistent with our results, Zhen *et al.* [10] demonstrated increased ALPL activity at 0.16–0.64 mM in rat primary osteoblasts. However, Patel *et al.* [13] found no differences at 0.1 mM, and Kasai *et al.* [14] reported reduced ALPL activity at 2 mM in mouse primary osteoblasts. Similar to Patel *et al.* [13], we did not detect differences in RUNX2 levels (at 0.1 mM), but unlike them, we revealed an increase in BGLAP production and a decrease in RANKL levels. Concentrations of BMP2 and VEGF could not be compared with published data, as these biomarkers have not been studied in primary osteoblast cell cultures yet. Nevertheless, VEGF and BMP2 are known to promote bone repair by stimulating both angiogenesis and osteogenesis and their upregulation may contribute to the beneficial effect of MET on fracture healing [19]. Kasai *et al.* [14] found no effect of MET at doses of 0.5 and 1 mM on matrix mineralization, which corresponds to our findings. However, these authors stated inhibited matrix mineralization, as well as gene expression of RUNX2, BGLAP, and IBSP at 2 mM. Taking all the results into consideration, we can conclude that MET at concentrations up to 1 mM can promote rat primary osteoblast viability, their differentiation and function, angiogenic signaling, and reduce osteoclastogenesis. A concentration of 100 mM was cytotoxic for osteoblast viability, while doses of 10 and 50 mM were already cytotoxic for matrix mineralization. In humans, MET overdose can lead to MET-associated lactic acidosis, which is correlated with worsening of acidosis and hyperlactatemia, and may negatively affect bone marrow [20]. In general, inhibited proliferation of bone marrow-derived multipotent mesenchymal stromal cells (BMSCs) and increased apoptosis have been reported after high-dose MET treatment [21]. Given that BMSCs serve as precursors for osteoblasts [22], excessive doses of MET may reduce the number of osteoblasts and also have an adverse effect on their function.

## Figures and Tables

**Fig. 1:**
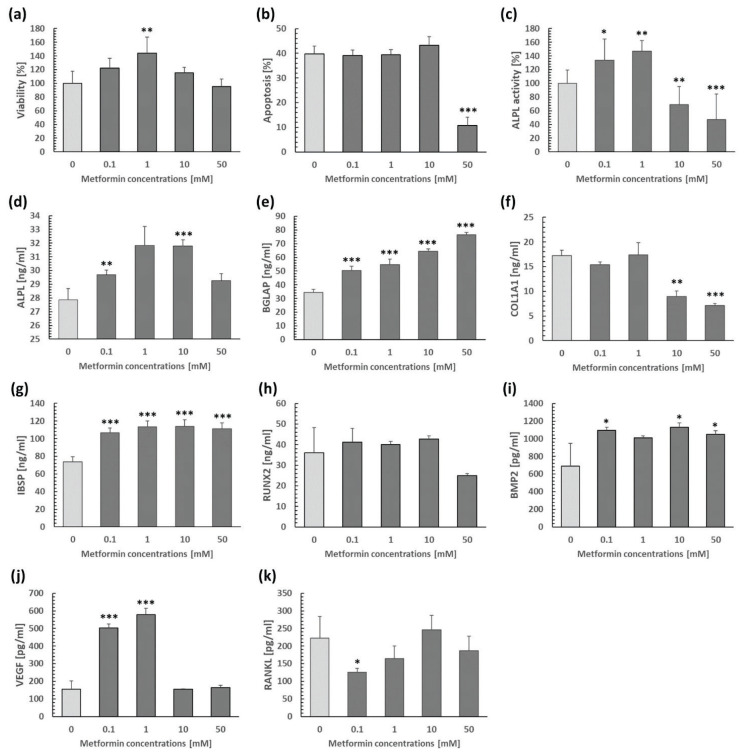
Osteoblast apoptosis (**a**), viability (**b**), ALPL activity (**c**), and the levels of ALPL (**d**), BGLAP (**e**), COL1A1 (**f**), IBSP (**g**), RUNX2 (**h**), BMP2 (**i**), VEGF (**j**) and RANKL (**k**) in cultured rat primary osteoblasts after MET administration at doses of 0–50 mM. * p<0.05, ** p<0.01, *** p<0.001, compared to the control group (0 mM).

**Fig. 2:**
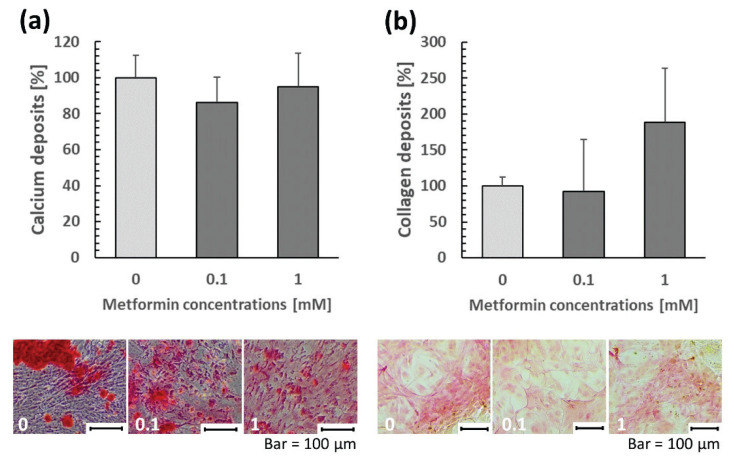
Deposition of calcium (**a**) and collagen (**b**) by cultured rat primary osteoblasts (measurements and representative microphotographs) after MET treatment at doses of 0.1 and 1 mM.

## References

[1] Sanchez-Rangel E, Inzucchi SE (2017). Metformin: clinical use in type 2 diabetes. Diabetologia.

[2] Blahova J, Martiniakova M, Babikova M, Kovacova V, Mondockova V, Omelka R (2021). Pharmaceutical Drugs and Natural Therapeutic Products for the Treatment of Type 2 Diabetes Mellitus. Pharmaceuticals.

[3] Zhao B, Luo J, Yu T, Zhou L, Lv H, Shang P (2020). Anticancer mechanisms of metformin: A review of the current evidence. Life Sci.

[4] Buczyńska A, Sidorkiewicz I, Krętowski AJ, Adamska A (2024). Examining the clinical relevance of metformin as an antioxidant intervention. Front Pharmacol.

[5] Mu W, Liang G, Feng Y, Jiang Y, Qu F (2022). The Potential Therapeutic Role of Metformin in Diabetic and Non-Diabetic Bone Impairment. Pharmaceuticals.

[6] Du D, Hong S, Zhang Y, Wang Z, Li H, Gao Z (2025). Metformin facilitates osteogenic differentiation of bone marrow stromal cells through AMPK-dependent autophagy: an investigation into the healing of osteoporotic fractures in murine models. J Orthop Surg Res.

[7] Yang K, Pei L, Zhou S, Tao L, Zhu Y (2021). Metformin attenuates H2O2-induced osteoblast apoptosis by regulating SIRT3 via the PI3K/AKT pathway. Exp Ther Med.

[8] Sun R, Liang C, Sun Y, Xu Y, Geng W, Li J (2022). Effects of metformin on the osteogenesis of alveolar BMSCs from diabetic patients and implant osseointegration in rats. Oral Diseases.

[9] Park SH, Kang MA, Moon YJ, Jang KY, Kim JR (2020). Metformin coordinates osteoblast/osteoclast differentiation associated with ischemic osteonecrosis. Aging.

[10] Zhen D, Chen Y, Tang X (2010). Metformin reverses the deleterious effects of high glucose on osteoblast function. J Diabetes Complications.

[11] Mai QG, Zhang ZM, Xu S, Lu M, Zhou RP, Zhao L, Jia CH (2011). Metformin stimulates osteoprotegerin and reduces RANKL expression in osteoblasts and ovariectomized rats. J Cell Biochem.

[12] Khan MP, Singh AK, Joharapurkar AA, Yadav M, Shree S, Kumar H, Gurjar A (2015). Pathophysiological Mechanism of Bone Loss in Type 2 Diabetes Involves Inverse Regulation of Osteoblast Function by PGC-1α and Skeletal Muscle Atrogenes: AdipoR1 as a Potential Target for Reversing Diabetes-Induced Osteopenia. Diabetes.

[13] Patel JJ, Butters OR, Arnett TR (2014). PPAR agonists stimulate adipogenesis at the expense of osteoblast differentiation while inhibiting osteoclast formation and activity. Cell Biochem Funct.

[14] Kasai T, Bandow K, Suzuki H, Chiba N, Kakimoto K, Ohnishi T, Kawamoto S (2009). Osteoblast differentiation is functionally associated with decreased AMP kinase activity. J Cell Physiol.

[15] Taylor SEB, Shah M, Orriss IR (2014). Generation of rodent and human osteoblasts. Bonekey Rep.

[16] Chava S, Chennakesavulu S, Gayatri BM, Reddy ABM (2018). A novel phosphorylation by AMP-activated kinase regulates RUNX2 from ubiquitination in osteogenesis over adipogenesis. Cell Death Dis.

[17] Duan W, Zou H, Zang N, Ma D, Yang B, Zhu L (2023). Metformin increases bone marrow adipose tissue by promoting mesenchymal stromal cells apoptosis. Aging.

[18] Lin J, Xu R, Shen X, Jiang H, Du S (2020). Metformin promotes the osseointegration of titanium implants under osteoporotic conditions by regulating BMSCs autophagy, and osteogenic differentiation. Biochem Biophys Res Commun.

[19] Ruan Z, Yin H, Wan TF, Lin ZR, Zhao SS, Long HT, Long C (2023). Metformin accelerates bone fracture healing by promoting type H vessel formation through inhibition of YAP1/TAZ expression. Bone Res.

[20] Hajsadeghi S, Gholizadeh Mesgarha M, Pour Mohammad A, Saberi Shahrbabaki A, Talebi A (2022). A concealed history behind the disaster: Extremely rare presentations of metformin toxicity in a patient with body dysmorphic disorder. Toxicol Rep.

[21] Śmieszek A, Czyrek A, Basinska K, Trynda J, Skaradzińska A, Siudzińska A, Marędziak M (2015). Effect of Metformin on Viability, Morphology, and Ultrastructure of Mouse Bone Marrow-Derived Multipotent Mesenchymal Stromal Cells and Balb/3T3 Embryonic Fibroblast Cell Line. Biomed Res Int.

[22] Gao Q, Wang L, Wang S, Huang B, Jing Y, Su J (2021). Bone Marrow Mesenchymal Stromal Cells: Identification, Classification, and Differentiation. Front Cell Dev Biol.

